# Patchwork Coating of Fragmented Ultra-Thin Films and Their Biomedical Applications in Burn Therapy and Antithrombotic Coating

**DOI:** 10.3390/ma8115404

**Published:** 2015-11-11

**Authors:** Yosuke Okamura, Yu Nagase, Shinji Takeoka

**Affiliations:** 1 Department of Applied Chemistry, School of Engineering, Tokai University, 4-1-1 Kitakaname, Hiratsuka, Kanagawa 259-1292, Japan; y.okamura@tokai-u.jp (Y.O.); yunagase@tokai-u.jp (Y.N.); 2 European Biomedical Science Institute, Organization for Regional and Inter-Regional Studies, Waseda University, 513 Wasedatsurumakicho, Shinjuku, Tokyo 162-0041, Japan; 3 Department of Life Science and Medical Bioscience, School of Advanced Science and Engineering, Waseda University, TWIns, 2-2 Wakamatsucho, Shinjuku, Tokyo 162-8480, Japan

**Keywords:** ultra-thin films (nanosheets), fragmentation, patchwork, biocompatibility, biodegradability, poly(l-lactic acid), phosphorylcholine

## Abstract

We have proposed free-standing centimeter-sized ultra-thin films (nanosheets) for biomedical applications. Such nanosheets exhibit unique properties such as transparency, flexibility, and good adhesiveness. However, they are only easily adhered to broad and flat surfaces due to their dimensions. To this end, we recently proposed an innovative nanomaterial: the nanosheets fragmented into submillimeter-size pieces. Intriguingly, such fragmented nanosheets could be adhered to uneven and irregular surfaces in addition to flat surfaces in a spread-out “patchwork” manner. We herein review the fabrication procedure and characterization of fragmented nanosheets composed of biodegradable polyesters and thermostable bio-friendly polymers, and their biomedical applications in burn therapy and antithrombotic coating using a “patchwork coating”.

## 1. Introduction

In the field of nanotechnology, free-standing ultra-thin films composed of organic materials, inorganic materials or their combination have been proposed as innovative two-dimensional nanomaterials [1,2,3]. Such films are often called nanosheets, nanofilms or nanomembranes due to their thickness of tens of nanometers. The nanosheets exhibit high transparency and flexibility as a result [3]. Over the years, a large number of fabrication procedures have been proposed; e.g., layer-by-layer deposition of the polyelectrolytes themselves or polyelectrolytes and inorganic materials with opposite net charges [4,5], Langmuir-Blodgett films of amphiphilic polymers [6], and a sol-gel process to synthesize interpenetrating polymer networks [7], polymerized lipid films [8,9], and so on. Using their structural features, large-surface-area nanosheets have been applied in a wide variety of fields, e.g., electrochemical studies and separation technologies such as sensors, energy storage, and selective adsorption.

We have recently proposed the use of bio-friendly nanosheets for biomedical applications [10,11,12,13,14,15,16,17]. Two types of bio-friendly polymers are discussed here. One is typical biodegradable polyesters such as poly(lactic acid) (PLLA) and its copolymers, *etc.* Such polymers have been clinically applied as implantable and biodegradable sutures [18] and bone screws [19] and so on. Therefore, it seems natural that the selection of biodegradable polyesters would be the shortest route to the clinical application of nanosheets. The other type is biocompatible polymers containing phosphorylcholine (PC) groups, which is one of the polar components of phospholipids in cell membranes [20]. The natural phospholipids themselves are definitely biocompatible. In terms of the workability of lipid moiety to films and coating materials, *etc.*, a synthetic polymer containing PC groups, 2-methacryloyloxyethyl phosphorylcholine polymer (so-called MPC polymer), was originally synthesized by Ishihara *et al.* [21,22]. The MPC polymers have high blood compatibility, resulting in the efficient reduction of the nonspecific adhesion of proteins and cells to the polymer surfaces. In order to provide thermal stability and adequate mechanical strength, we have synthesized diamine and diol aromatic monomers containing the PC groups, from which various aromatic polymers such as polyamides, polyimides, poly(urethane-urea)s, polyesters, and polyurethanes were synthesized by polycondensation and polyaddition [21,22,23,24,25]. In fact, we have reported that these polymers also exhibit high thermal stability and good mechanical properties as well as good compatibility against blood [23,24,25,26,27].

In this review, we describe the fundamental features of centimeter-sized bio-friendly nanosheets composed of biodegradable polyesters (PLLA), biocompatible polyimides, and segmented polyurethanes containing PC groups developed in our groups (named as PIPC and SPUPC, respectively). We next focus on the submillimeter-sized nanosheets fragmented by a homogenizer. In particular, we introduce the fabrication procedure, their properties, and biomedical applications as aqueous surface modifiers in burn therapy and antithrombotic coatings. The fragmented PLLA nanosheets would be suitable for the former application because they degrade gradually on the burn wound. The fragmented PIPC and SPUPC nanosheets would be suitable for the latter application to sterilize medical devices exposed continuously to blood such as catheters and artificial organs, *etc.*

## 2. Fabrication and Characterization of Centimeter-Sized Nanosheets

### 2.1. Biodegradable Nanosheets

**Figure 1:**
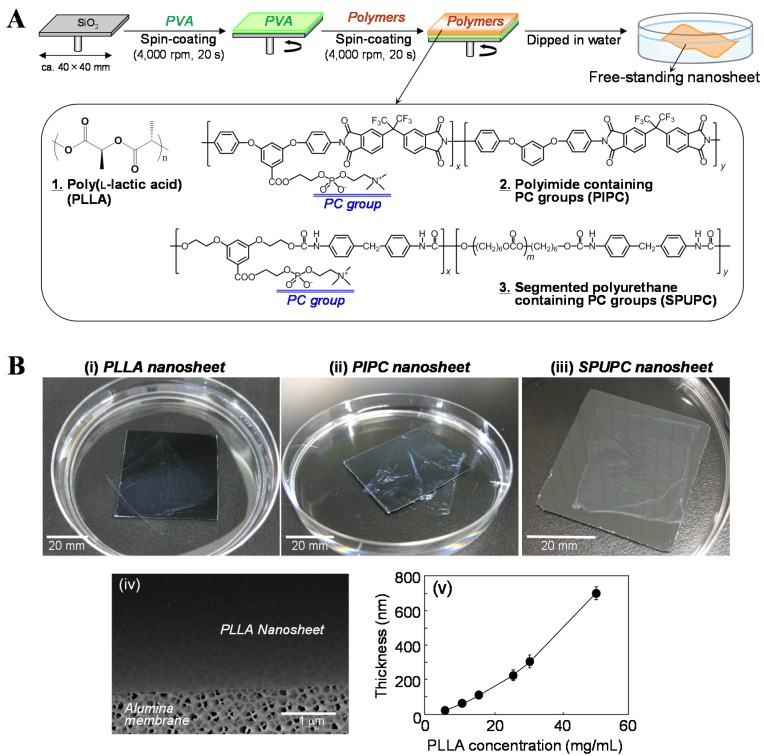
Free-standing centimeter-sized nanosheets composed of bio-friendly polymers. (**A**) Fabrication scheme for the nanosheet using a water-soluble sacrificial layer, and chemical structures of the bio-friendly polymers utilized in this review (obtained from [16] and partially modified, with permission from Trans. Mat. Res. Soc. Japan.); (**B**) Macroscopic images of the free-standing nanosheets of (**i**) PLLA; (**ii**) PIPC; and (**iii**) SPUPC exfoliated in water (originally photographed according to [13,15,16,17]); (**iv**) SEM image of the surface of the PLLA nanosheet adhered to an alumina membrane (originally photographed according to [13]); (**v**) Relationship of thickness of the PLLA nanosheet and PLLA concentration in a spin-coating process (obtained from [13] and partially modified, with permission from John Wiley & Sons.).

We have proposed the development of free-standing centimeter-sized nanosheets composed of biodegradable polymers, which can be easily detached from substrates using a sacrificial layer (Figure 1A) [13,15,16,17]. The key point in this approach is to utilize the difference in solubility between the sacrificial layers and the nanosheets. The sacrificial layer is soluble in appropriate solvents which do not dissolve the nanosheets. At the same time, the sacrificial layer is insoluble in the solvents that dissolve the polymers making up the nanosheets. For instance, poly(vinyl alcohol) (PVA) is soluble in water, but insoluble in halogenated solvents such as methylene chloride and chloroform which are used to prepare nanosheets. In contrast, poly(l-lactic acid) (PLLA), which is a typical biodegradable polymer, is soluble in halogenated solvents but is insoluble in water. PVA thus acts as a water-soluble sacrificial layer to fabricate the free-standing nanosheets composed of PLLA into water [13,15,16,17]. The detailed fabrication procedure for the free-standing PLLA nanosheets can be summarized as follows. First of all, an aqueous solution of 10 mg/mL PVA was dropped onto a SiO_2_ substrate with a smooth surface. The substrate was spin-coated at 4000 rpm for 20 s and dried at 70 °C. A methylene chloride solution of 5 mg/mL PLLA was then spin-coated onto the PVA-coated substrates under the same spin-coating conditions. When the substrate was immersed into water, a PLLA nanosheet was easily exfoliated from the substrate by dissolving only the PVA sacrificial layer in water (Figure 1B(i)). The obtained nanosheets were transparent and were very flexible. The thickness of the PLLA nanosheet was approximately 20 nm and the surface roughness was measured to be a few nanometers. We confirmed that the PLLA nanosheet scooped onto an alumina membrane had a flat surface without any cracks using a scanning electron microscope (SEM) (Figure 1B(iv)). Furthermore, we demonstrated that the thickness of the nanosheet was controlled by the concentration of the polymer solution used in the spin-coating process (Figure 1B(v)) [13]. This fabrication procedure can be adapted to other polymers, e.g., versatile polymers such as polystyrene and poly(methyl methacrylate), *etc.*, and biodegradable polyesters such as copolymers of lactide and glycolide and polycaprolactone, *etc.* Free-standing polymer nanosheets fabricated by layer-by-layer and Langmuir-Blodgett processes have been proposed as described in the Introduction [4,6]. These techniques are very useful to freely control the thickness and surface properties of the nanosheets at a molecular level due to the stepwise adsorption of the monolayer via secondary interactions such as electrostatic interaction, hydrogen bonding, and hydrophobic interaction, *etc.* [4,6]. However, these processes take a long time to fabricate the nanosheet. On the other hand, our methodology as shown above has an advantage in the reduction of fabrication time (only several minutes) if it has satisfied the condition to utilize the difference in solubility between the sacrificial layers and the nanosheets.

Basic properties of PLLA were also summarized as follows. Our differential scanning calorimetric analysis revealed that the grass transition and melting temperatures of PLLA were 53 °C and 166 °C, respectively. This means that the thermal stability of PLLA is, of course, lower than that of aromatic polymers such as PIPC and SPUPC, as described below. However, PLLA is well known as a representative biodegradable polymer unlike PIPC and SPUPC. In fact, a PLLA nanosheet with a thickness of 20 nm was degraded within one week under enzymatic conditions as reported previously [13].

### 2.2. PC-Containing Polymer Nanosheets

We have synthesized a series of biocompatible aromatic polymers containing PC groups to provide thermostability and robust mechanical properties [23,24,25,26,27]. In fact, polyimides, polyamides, poly(urethane-urea)s, polyesters, and segmented polyurethanes have been synthesized by polycondensation or polyaddition of diamine and diol aromatic monomers introducing PC groups [23,24,25,26,27]. We focus here on PIPC with a PC content of 14 mol% and SPUPC with that of 34 mol%. The thermal stabilities of the obtained PIPC and SPUPC were measured by thermal gravimetric analysis (TGA). The weight loss of the PIPC and SPUPC started at around the same temperature (*ca.* 220 °C). On the other hand, the initial temperatures of decomposition were lower than those of control PI and SPU without PC groups (*ca.* 450 °C, *ca.* 300 °C, respectively). These results indicate that the thermal decomposition of PIPC and SPUPC is initiated by the degradation of PC groups. This phenomenon is similar to the properties of other PC-containing polymers reported previously [27]. However, thermal stabilities of these polymers are still acceptable, being around 200 °C. Therefore, these polymers would tolerate a sterilization process of over 150 °C.

We next tried to fabricate free-standing nanosheets composed of PIPC and SPUPC. These polymers were also dissolved in methylene chloride, adjusting the concentration to 10 mg/mL. Using PVA as a sacrificial layer as described above, we have succeeded in the fabrication of the PIPC and SPUPC nanosheets (Figure 1B(ii),(iii)). The thickness of each nanosheet was measured to be approximately 40 nm and 66 nm, respectively. These nanosheets were also transparent and very flexible in nature. Intriguingly, the size of the obtained PIPC nanosheets was equivalent to that of the SiO_2_ substrate, as was found for the PLLA nanosheets, whereas the SPUPC nanosheets shrunk after being detached from the substrates (Figure 1B(ii),(iii)). This can be explained as follows: SPUPC nanosheets would be extended on the substrates by the centrifugal force produced in the spin-coating process. When detached from the substrate, the nanosheets shrank due to their elasticity. This phenomenon was not observed in the nanosheets composed of non-elastic polymers such as PIPC and PLLA.

### 2.3. Unique Property of the Nanosheets: High Adhesion

When typical cast films with a thickness of over several μm are attached to various surfaces such as plastics, glasses, steels, and physiological tissues, *etc.*, they are easily detached. Intriguingly, it is difficult to detach the nanosheets dried on these surfaces, even when no adhesive agents have been employed. To this end, we quantitatively measured the relationship between the adhesive strength of the nanosheets and their thickness using a micro-scratch tester [28], where the nanosheets adhered on the SiO_2_ substrate were scratched with a diamond stylus [13]. We can summarize the results on adhesiveness of the PLLA nanosheets as follows. The load value just after detaching the PLLA nanosheet with a thickness of approximately 20 nm was measured to be (1.7 ± 0.3) × 10^5^ N/m [13]. This value was similar to that of the nanosheet with 60 nm thickness. However, the values of the nanosheets with the thickness of approximately 100, 230, 310, and 400 nm drastically decreased to (0.80 ± 0.1) × 10^5^, (0.69 ± 0.1) × 10^5^, (0.76 ± 0.1) × 10^5^, and (0.45 ± 0.1) × 10^5^ N/m, respectively [13]. This can be explained as follows: the nanosheets with a thickness of less than 100 nm possess a flexible structure and the roughness of their surface was quite low. When the nanosheets were attached to the various surfaces, they could conform to the roughness of the surfaces via surface-contact interactions. We have confirmed that this tendency was equivalent for other components of the nanosheets such as polystyrene, copolymers of lactide and glycolide, PIPC and SPUPC, *etc.* For instance, the load values of the PIPC nanosheets were similar to those of PLLA nanosheets (thickness of 40 nm: (1.4 ± 0.4) × 10^5^ N/m, thickness of 160 nm: (0.77 ± 0.2) × 10^5^ N/m, thickness of 420 nm: (0.41 ± 0.2) × 10^5^ N/m) [17]. However, the load values of the SPUPC nanosheets were significantly higher than those of the PLLA and PIPC nanosheets (thickness of 35 nm: (7.0 ± 2.8) × 10^5^ N/m, thickness of 150 nm: (2.0 ± 0.2) × 10^5^ N/m, thickness of 510 nm: (0.46 ± 0.1) × 10^5^ N/m) [27]. The elastic modulus of the elastic bulk SPUPC was measured to be 23.5 MPa [27]. On the other hand, the elastic moduli of bulk PLLA and PIPC, which are non-elastic polymers, were reported to be several GPa [17,29]. This result suggests that the elasticity of the nanosheets would be involved in their adhesive strength to the surfaces. We thus demonstrated that the unique property of these nano-thickness films was their high potential to adhere to several different surfaces.

## 3. Fabrication and Characterization of Submillimeter-Sized Nanosheets (Fragmented Nanosheets)

### 3.1. Fabrication of the Fragmented Nanosheets

**Figure 2:**
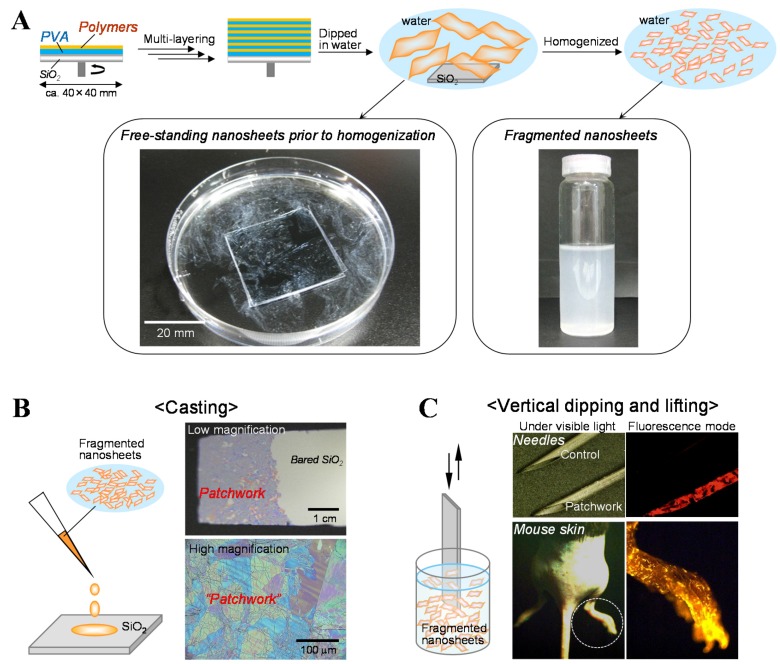
Fragmented submillimeter-sized nanosheets composed of bio-friendly polymers. (**A**) Scheme of the fragmented nanosheets (obtained from [14] and partially modified, with permission from John Wiley & Sons; obtained from [16] and partially modified, with permission from Trans. Mat. Res. Soc. Japan.) and macroscopic images of the PLLA nanosheets suspended in water before (**left**) and after (**right**) homogenization (originally photographed according to [14]); (**B**) Casting method of the patchwork coating of the fragmented PLLA nanosheets on a SiO_2_ substrate (schematic image was obtained from [16] and partially modified, and photographs were originally taken according to [14]); (**C**) Vertical dipping and lifting method of patchwork coating of the PLLA nanosheets on a needle and on mouse skin, including toes (originally photographed according to [14]).

We summarized the unique properties of centimeter-sized nanosheets, which possess high adhesiveness, flexibility, and transparency in Section 2. However, these nanosheets can only adapt to be attached to comparatively broad and flat surfaces due to their relatively large size. In this section, we introduce a simple fabrication procedure for submillimeter-sized nanosheets, designated as fragmented nanosheets, for coating onto irregular and uneven surfaces, as well as broad and flat surfaces. First of all, we developed a large-scale preparation of centimeter-sized PLLA nanosheets by a multi-layering technique as follows [14,15,16]. As depicted in Figure 2A, an aqueous solution of 100 mg/mL PVA was spin-coated as a sacrificial layer on a SiO_2_ substrate at 4000 rpm for 20 s and dried at 70 °C [14,15,16]. A methylene chloride solution of 10 mg/mL PLLA was then spin-coated and dried on the PVA-coated substrate under the same conditions, corresponding to the thickness of approximately 60 nm. The multi-layering and drying of PVA and PLLA were performed dozens of times. As shown in Figure 2A, abundant centimeter-sized PLLA nanosheets were collected by immersion into water, corresponding to the number of multi-layering processes. The surface area of each nanosheet was approximately 1600 mm^2^, corresponding to the size of the SiO_2_ substrate (40 × 40 mm). Subsequently, when the nanosheets were homogenized at 30,000 rpm, they were quickly and discretely fragmented in water. Intriguingly, the obtained fragmented nanosheets were homogeneously suspended in water and their viscosity of the dispersion was increased (Figure 2A). Furthermore, the surface area of each fragmented nanosheet became 0.24 ± 0.08 mm^2^ 7 min after homogenization, corresponding to the area of less than one-thousandth of the centimeter-sized nanosheets present prior to homogenization [14]. Using this simple methodology, we could prepare fragmented nanosheets composed of PIPC (surface area of each nanosheet: 6800 ± 208 μm^2^, thickness: 42 nm) and SPUPC (surface area of each nanosheet: 3900 ± 1300 μm^2^, thickness: 66 nm) [17]. These fragmented nanosheets can be utilized as a suspension in water. Moreover, they have a unique size aspect ratio of submillimeters in size but only tens of nanometers in thickness. Therefore, the fragmented nanosheets would be included in innovative anisotropic nanomaterials as proposed previously, e.g., inorganic nanosheets composed of graphene dissociated from graphite [30,31] and anisotropic polymer particles (discs- and snowman-like shapes, *etc.*) fabricated by seeded dispersion polymerization [32,33].

### 3.2. Unique Adhesion Behavior of the Fragmented Nanosheets

The fragmented nanosheets composed of PLLA were cast and dried on the flat SiO_2_ substrate as illustrated in Figure 2B [16]. Interestingly, the nanosheets were firmly adhered to the substrate in a spread-out configuration (no curled or aggregated configurations of the sheets were observed) [14]. The adhesion behavior resembles “patchwork” or “stained-glass” based on the structural colors of the nanosheets adhered on the substrate (Figure 2B) [14]. Moreover, we have demonstrated that the nanosheets were able to adhere not only to a flat substrate but also to irregular and uneven surfaces by casting and dipping processes. In fact, when various surfaces such as a needle and a mouse body, including toes, were vertically dipped into a suspension of fluorescent-labeled nanosheets and lifted up, they were effectively coated with the fluorescent nanosheets (Figure 2C) [14]. It is also interesting that the adhesion was invisible in a visible light due to the nanometer-scaled thickness adhesion. Furthermore, the nanosheets were difficult to detach from the surfaces, even by being dipped in water again and being scratched with tweezers, once they dried onto the surfaces. This suggests that the nanosheets were adhered to the surfaces and to each other via surface-contact interactions. We called this adhesion of the fragmented nanosheets a “patchwork coating”. From these results, we were inspired to investigate whether such “patchwork coating” could be used as aqueous surface modifiers, as described in the next section.

### 3.3. Biomedical Applications of the Fragmented Nanosheets as Aqueous Surface Modifiers

#### 3.3.1. Burn Therapy

A burn trauma of skin or tissues is caused by exposure to heat, chemicals, or electricity. An important issue in burn therapy is to decrease the possibility of infection in burn wounds during healing as soon as possible [34]. Otherwise, superficial burn wounds may advance to cause deeper tissue damage, resulting in severe sepsis in a worst-case situation. To this end, several types of wound dressings have been clinically applied for burn therapy [35]. Such dressings with a quite thick structure are easy to adhere to flat and broad surfaces such as the back and belly, *etc.* However, it is difficult to adhere large nanosheets to burn wounds with an irregular or uneven shape, e.g., fingers and toes, *etc.*, as can be imagined. In this section, we introduce an application for burn therapy using a “patchwork coating” of fragmented nanosheets, which acts as an aqueous surface modifier against burn wounds [14].

**Figure 3:**
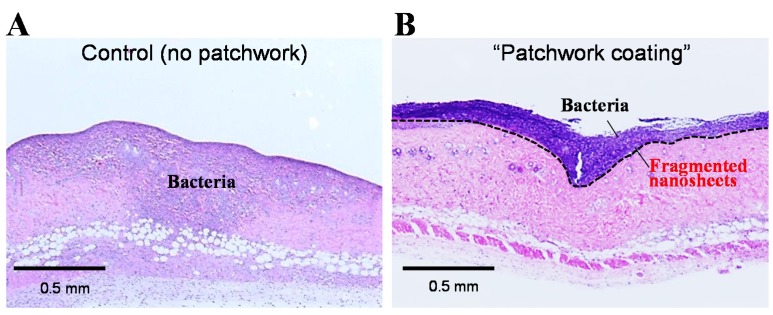
Prevention of infection using a patchwork coating of fragmented PLLA nanosheets in a burn wound model in mice. (**A**) Histological image of skin in control experiments without patchwork coating. The bacteria (*Pseudomonas* *aeruginosa*) penetrated into the dermis and subcutaneous layer, resulting in infection; (**B**) Histological image of skin treated with patchwork-like coating. The bacteria remained on the surface of the skin (dotted line), showing the prevention of infection (obtained from [16], and partially modified, with permission from Trans. Mat. Res. Soc. Japan.).

According to a previous protocol in a mouse model [35], we prepared the burn wound on which the fragmented nanosheets composed of PLLA were added and dried to provide a “patchwork coating”. Bacteria of *Pseudomonas aeruginosa* was then added to the patchwork region. As a negative control, the same bacteria was added to a bare burn wound without a patchwork coating. Each skin was removed from the mice three days after treatment and stained with Hematoxylin-Eosin for histological observation. In the negative control, the bacteria migrated into both the dermis and the subcutaneous layer (Figure 3A) [16]. This means that the burn wound was infected with the bacteria due to the defective dermis. In the case of the “patchwork coating”, the bacteria stained in blue-purple did not penetrate into the dermis, but remained on the dermis (Figure 3B) [16]. The “patchwork coating” may thus constitute an alternative to conventional burn therapies to prevent infection. In other words, we could say that the “patchwork coating” acts as an aqueous surface modifier to alter the surface properties of infection-susceptible burn wounds. Moreover, it is also noteworthy that a surgeon can directly look at conditions of the wound even after “patchwork coating” due to the transparent nano-coating. This would be only achievable by “patchwork coating” of the nanosheets because the clinically applied wound dressings composed of carboxymethyl cellulose and gelatin [36] or alginate films [37] are opaque films.

#### 3.3.2. Antithrombotic Coating

Blood consists of three kinds of floating cells: erythrocytes, leukocytes, and thrombocytes (often called platelets). Among their functions, platelets are involved in pathological thrombosis in addition to normal hemostasis [38]. An important point for the application of blood-compatible materials is to avoid unexpected thrombosis triggered by the interactions between platelets and the surface of the implanted materials. As noted above, we succeeded in the preparation of fragmented nanosheets composed of PIPC and SPUPC containing PC groups. Here we describe an aqueous antithrombotic coating using a “patchwork coating” of these materials.

**Figure 4:**
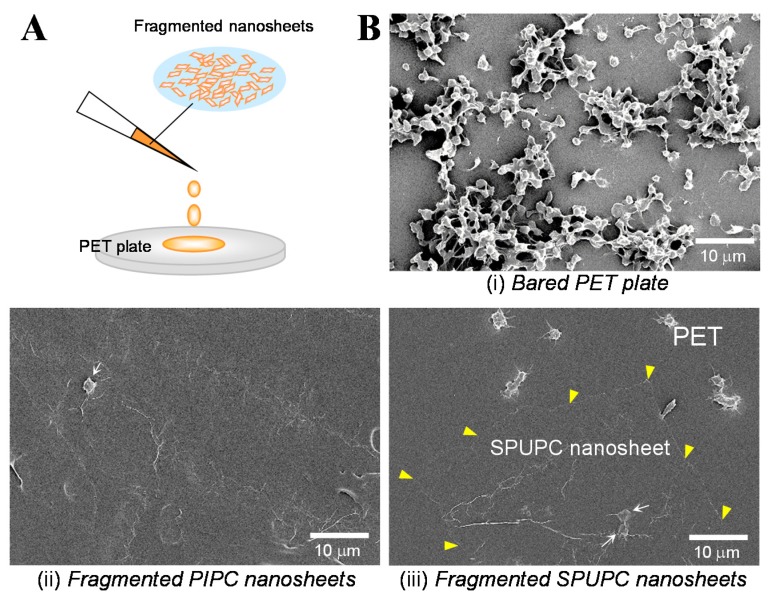
Patchwork coating of the fragmented PIPC and SPUPC nanosheets to demonstrate excellent blood compatibility. (**A**) Schematic image of patchwork coating to the PET plates ( obtained from [16] and partially modified, with permission from Trans. Mat. Res. Soc. Japan.); (**B**) Platelet adhesion test. SEM images of (**i**) bare PET plate, patchwork coating of fragmented nanosheets of (**ii**) PIPC and (**iii**) SPUPC, to which platelet-rich plasma was dropped and incubated at 37 °C for 2 h. Arrows in (**ii**) and (**iii**) show the adhered platelets on the nanosheets, and yellow triangles in (**iii**) show the fragmented SPUPC nanosheets on the PET plate (originally photographed according to [16,17]).

We prepared the fragmented PIPC and SPUPC nanosheets, which were uniformly suspended in water as described in Section 3.1. When the fragmented nanosheets were dropped and dried on a poly(ethylene terephthalate) (PET) plate as a model surface, they formed a “patchwork coating” in a similar fashion to the fragmented PLLA nanosheets (Figure 4A). We next evaluated the blood compatibility of the PET plates before and after patchwork. Actually, platelet-rich plasma donated from healthy volunteers was added to the plates, followed by an incubation at 37 °C for 2 h. In a control (without patchwork), abundant platelets were non-specifically activated at filopodial extension state, and adhered and aggregated on the bare PET plates (Figure 4B(i)) [16,17]. In cases of the patchwork-coating, few platelets were indeed adhered on the surfaces, although some wrinkles of the patchwork were observed (Figure 4B(ii),(iii)) [16,17]. These results indicate that the inhibitory effect of platelet adhesion was a PC group-dependent phenomenon, which was comparable to the results reported previously [39,40]. Consequently, we demonstrated that a “patchwork coating” of the fragmented PC-containing nanosheets acts as an aqueous surface modifier to provide an antithrombotic property. Furthermore, the nanosheets may be applied to surfaces of medical devices such as catheters and artificial organs and so on.

## 4. Summary

In this review, we have described the preparation and use of fragmented bio-friendly nanosheets, using a simple fabrication process consisting of a spin-coating, a novel peeling technique, and a fragmentation procedure. The fragmented nanosheets acted as aqueous surface modifiers to effectively coat even, irregular, and uneven surfaces in a patchwork adhesion manner. For their biomedical applications, we demonstrated that such a coating technique has the potential to protect burn wounds from the infection of bacteria and to provide blood compatibility to various surfaces. We expect that these nanosheets with their unique properties possess high potential as innovative nanomaterials for advanced applications in various research fields, e.g., energy and environmental sciences in addition to medicine.
